# The Potential of Mobile Apps for Improving Asthma Self-Management: A Review of Publicly Available and Well-Adopted Asthma Apps

**DOI:** 10.2196/mhealth.7177

**Published:** 2017-08-02

**Authors:** Peter Tinschert, Robert Jakob, Filipe Barata, Jan-Niklas Kramer, Tobias Kowatsch

**Affiliations:** ^1^ Center for Digital Health Interventions Institute of Technology Management (ITEM-HSG) University of St. Gallen St. Gallen Switzerland; ^2^ Operations & Supply Chain Management School of Management Technical University of Munich Munich Germany; ^3^ Center for Digital Health Interventions Department for Management, Technology and Economics ETH Zurich Zurich Switzerland

**Keywords:** asthma, self care, disease management, mobile applications, smartphone, mHealth, eHealth, mobile health, behavior and behavior mechanisms, review

## Abstract

**Background:**

Effective disease self-management lowers asthma’s burden of disease for both individual patients and health care systems. In principle, mobile health (mHealth) apps could enable effective asthma self-management interventions that improve a patient’s quality of life while simultaneously reducing the overall treatment costs for health care systems. However, prior reviews in this field have found that mHealth apps for asthma lack clinical evaluation and are often not based on medical guidelines. Yet, beyond the missing evidence for clinical efficacy, little is known about the potential apps might have for improving asthma self-management.

**Objective:**

The aim of this study was to assess the potential of publicly available and well-adopted mHealth apps for improving asthma self-management.

**Methods:**

The Apple App store and Google Play store were systematically searched for asthma apps. In total, 523 apps were identified, of which 38 apps matched the selection criteria to be included in the review. Four requirements of app potential were investigated: app functions, potential to change behavior (by means of a behavior change technique taxonomy), potential to promote app use (by means of a gamification components taxonomy), and app quality (by means of the Mobile Application Rating Scale [MARS]).

**Results:**

The most commonly implemented functions in the 38 reviewed asthma apps were tracking (30/38, 79%) and information (26/38, 68%) functions, followed by assessment (20/38, 53%) and notification (18/38, 47%) functions. On average, the reviewed apps applied 7.12 of 26 available behavior change techniques (standard deviation [SD]=4.46) and 4.89 of 31 available gamification components (SD=4.21). Average app quality was acceptable (mean=3.17/5, SD=0.58), whereas subjective app quality lied between poor and acceptable (mean=2.65/5, SD=0.87). Additionally, the sum scores of all review frameworks were significantly correlated (lowest correlation: *r*_36_=.33, *P*=.04 between number of functions and gamification components; highest correlation: *r*_36_=.80, *P*<.001 between number of behavior change techniques and gamification components), which suggests that an app’s potential tends to be consistent across review frameworks.

**Conclusions:**

Several apps were identified that performed consistently well across all applied review frameworks, thus indicating the potential mHealth apps offer for improving asthma self-management. However, many apps suffer from low quality. Therefore, app reviews should be considered as a decision support tool before deciding which app to integrate into a patient’s asthma self-management. Furthermore, several research-practice gaps were identified that app developers should consider addressing in future asthma apps.

## Introduction

Asthma, a chronic airway disease characterized by respiratory symptoms, affects an estimated 235 million [1] to 334 million [2] people worldwide. In the United States alone, 24 million people suffer from asthma (ie, 8.6% of children and 7.4% of adults; [3]). Due to its high prevalence and ongoing treatment throughout the lifetime of most patients, asthma costs the US health care system around US 56 billion dollars annually [4].

Health information technology can reduce the burden of chronic diseases such as asthma for patients and health care systems [5,6]. Mobile health (mHealth) apps in particular could enable low-cost and clinically efficacious interventions for asthma [7]. With virtually unlimited scalability and availability, apps could empower patients in their everyday asthma self-management by delivering evidence-based interventions. Research has shown that such interventions (eg, dissemination of educational materials and symptom monitoring tools) improve a patient’s quality of life and limit excess health care utilization [8]. Moreover, data obtained through mobile phone sensors and connected medical devices (eg, smart inhalers) can be used to deliver self-management interventions tailored to the specific needs of patients, thus increasing the intervention’s efficacy [9,10].

However, whereas first studies have indicated that asthma apps can indeed be effective tools for supporting patients in their self-management [11,12], reviews of asthma apps come to either ambiguous [13] or rather disappointing conclusions regarding the value of apps for improving asthma self-management [14,15]. These reviews have based their conclusions mainly on a lack of methodologically sound studies [13] and the limited application of medical guidelines [14,15].

Although an evidence base is highly desirable for health apps in general, it is unrealistic to expect app developers to conduct clinical studies or to hire clinical experts as consultants. Reasons for this are, among others, the high costs and difficulty of clinical research [16]. Additionally, app stores do not require developers to indicate the evidence base of an app (eg, Apple App store [17]). mHealth apps are also often not comprehensively regulated by public authorities (eg, in the United States, the Food and Drug Administration only regulates a subset of the available apps [18]). Even though patients tend to consider the evidence base as an important selection criterion for mHealth apps [19], they are often not able to make an informed judgment regarding such characteristics of an app [20]. All these factors contribute to the fact that patients keep using publicly available asthma apps regardless of missing validation studies or an app’s limited application of medical guidelines.

Therefore, it is necessary for the clinical practice to develop a more comprehensive understanding of the value that publicly available asthma apps might offer for asthmatics. Research pursuing this objective would need to address the limitations of the aforementioned reviews, which have focused exclusively on peer-reviewed papers and not publicly available apps [13] or on qualitative analysis of app content without applying review frameworks with quantifiable results [14,15].

To overcome these shortcomings, the present review applies review frameworks (eg, behavior change technique and gamification taxonomies [21,22]) and thereby, quantifies the characteristics of asthma apps. By making use of such frameworks, this review investigates the potential of well-adopted asthma apps for supporting asthmatics, even if clinical evidence is not available. In the next section, a functional definition of the term “app potential” is provided. The definition consists of specific requirements that an asthma app should fulfill to enable a positive effect on a patient’s asthma self-management.

In conclusion, this app review assesses the potential of publicly available and well-adopted asthma apps for supporting patients in their disease management. Furthermore, research-practice gaps will be identified in hopes of stimulating the development of new and more sophisticated asthma apps.

## Methods

### Overview

In this review, an app’s potential to support patients in their disease management is defined as the degree to which an app fulfills the following four requirements: (R1) an app has to contain some kind of “active ingredient,” that is, functions supporting patients in their disease management; (R2) similar to traditional asthma self-management programs offered by health care entities [23], an app should be able to change or guide behavior relevant for effective asthma self-management; (R3) an app should motivate the patient to use the app to deliver the active ingredients; and (R4) an app has to be of adequate quality (eg, functionality and aesthetics of an app) to guarantee a sufficient implementation quality of the other postulated requirements.

More specifically, this review addresses (R1) by reviewing available functions (similar to [14,15]), (R2) by applying a taxonomy of behavior change techniques [21], (R3) by rating a taxonomy of gamification components [22], and (R4) by using the Mobile Application Rating Scale (MARS; [24]).

### Systematic Search

**Figure 1:**
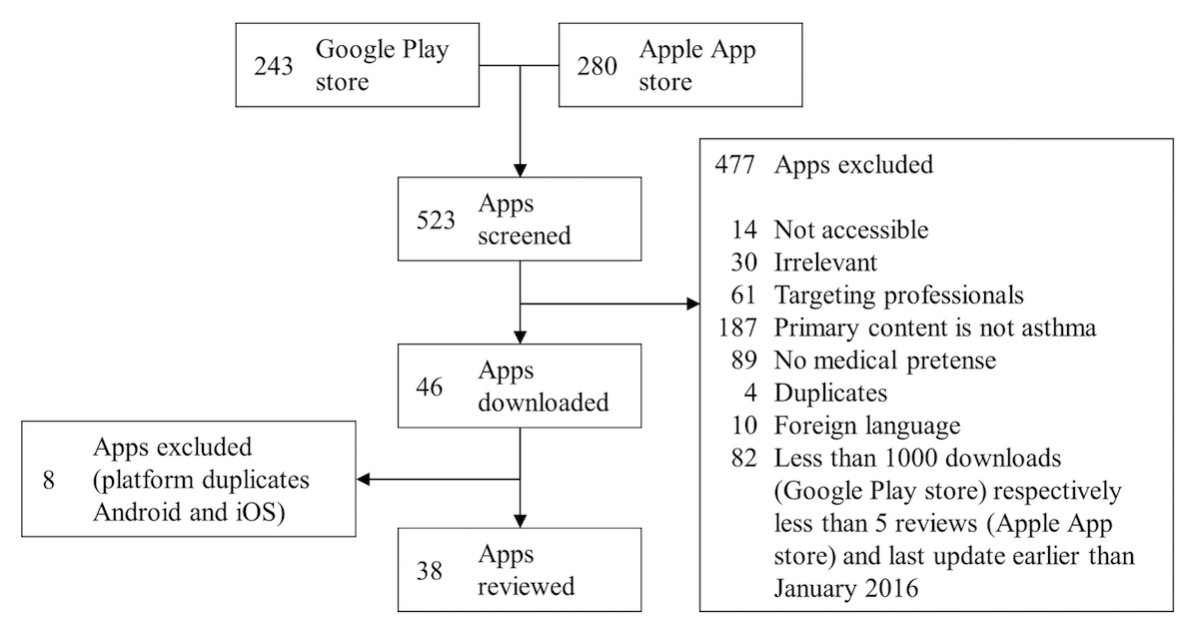
Systematic search and exclusion criteria.

The second author (RJ) searched the term “asthma” in the US versions of the two most popular app stores: Google Play store and Apple App store [25]. The latest app search was conducted on April 10, 2016. In total, 523 apps were identified (Figure 1).

### Inclusion and Exclusion Criteria

The strategy for app selection was to include publicly available and well-adopted apps that are designed specifically for asthma. To identify these apps, the following exclusion criteria were applied: no access (eg, only accessible through a special health program), irrelevance (ie, apps completely unrelated to asthma), target group are professionals (eg, apps supporting physicians), primary content is not asthma (eg, general weather or nutrition apps with very limited ancillary functions dedicated to asthmatics), no medical pretense (eg, yoga and acupressure apps), duplicates (ie, different versions of the same app with marginally different functions), and foreign language (ie, apps not in English). To ensure that relatively well-adopted apps are reviewed, apps with less than 1000 downloads (Google Play store) or less than five reviews (Apple App store) were excluded.

Because the number of downloads or reviews of an app depends on its release date, apps with less than 1000 downloads (Google Play store) or five reviews (Apple App store) were not excluded if they were updated in 2016. Otherwise, a selection bias against recently released apps would have occurred. In fact, ten otherwise excluded apps remained in this review due to this criterion (ie, four Android and six iPhone operating system [iOS] apps).

Based on the specified criteria, 477 apps were excluded. The remaining 46 apps were checked for duplicates between the two platforms (ie, Android and iOS). After excluding eight duplicates, the systematic search yielded 38 apps to review.

### Review Process

The review process consisted of two main steps. First, basic information about the app was gathered from the corresponding app store (eg, app description, date of last update, developer, number of downloads if available, number of user reviews, and average rating). Only publicly available data, which was directly extracted from the app stores, was used in this process. Additionally, an app’s website was consulted if available.

Second, the four requirements for app potential were evaluated. Except for the MARS framework, for which multiple mobile phones and tablets were used (ie, Huawei P9 Lite, HTC M9, LG Leon 4G LTE, iPad Air 2, and iPhone 6), apps were reviewed on a LG Leon 4G LTE (Android) and iPhone 6 (iOS). In the next section, the measures of the four app potential requirements are introduced in more detail.

### Measures of App Potential Requirements

#### R1: App Functions

An exhaustive list of all existing functions was developed through a bottom-up review approach: whenever a function was encountered that was not yet specified in the list, it was expanded accordingly. In this way, a total of 42 functions was identified. Afterwards, the available functions were assessed in all apps based on the complete list.

Additionally, functions were grouped post hoc into four categories: tracking (eg, of peak flow values), information (eg, about asthma symptoms), assessment (eg, indicating asthma control by color-coding peak flow values), and notification functions (eg, medication intake reminder). These function categories are conceptually identical to prior app reviews [14,15], except for one difference: the category of therapeutic tools was replaced with notification functions. Reasons for this are the low prevalence of therapeutic tools in asthma apps [15] and the potential utility of notification functions for asthma self-management (eg, as reminders for physician appointments [26]).

#### R2: Potential to Change Behavior

Many reviews have applied behavior change technique taxonomies as measures of behavior change efforts in health interventions and apps (eg, [27-29]). However, to the best of the authors’ knowledge, they have not yet been applied to the domain of asthma apps.

In this review, the application of the 26 behavior change techniques proposed by Abraham and Michie [21] was rated for each app. Ratings were dummy coded (1=“technique fully applied”, 0=“technique not applied”). The first and second author (PT and RJ) agreed on explicit rating criteria (see Multimedia Appendix 1 for an exemplary criteria list) and related functions for all behavior change techniques (eg, social network functions enable the application of the behavior change techniques “provide information about other’s approval,” “provide opportunities for social comparison,” and “plan social support or social change”). This approach addressed the issue of mediocre interrater reliability for some of the behavior change techniques (ie, whereas the mean kappa values ranged from .74 to .82 in the original publication, 20 out of 71 kappa scores (28%) were below .70 [21]). By applying explicit and objective rating criteria, the process of rating behavior change techniques was facilitated, and rating ambiguity was reduced. Differences in ratings between the first and second author were resolved in discussions.

In some instances, a behavior change technique was rated as partially applied (.50). This was the case if an app fulfilled the application criteria only partially (eg, nonadjustable task reminder for “time management”) or if an agreement between the first and second author could not be reached.

#### R3: Potential to Promote App Use

Gamification is a promising way to promote the use of information technology (IT) systems such as mobile apps [22,30]. A taxonomy of 31 gamification components [22] was applied to rate an app’s potential for motivating app use. Similar to the rating process of behavior change techniques, the first and second author agreed on an explicit list of relevant rating criteria and related functions for gamification components (see Multimedia Appendix 2 for an exemplary criteria list). Again, dummy coding was used for rating gamification components and partially implemented components were considered (1=“gamification component fully applied,” .50=“gamification component partially applied,” and 0=“gamification component not applied”). Differences in ratings were resolved in discussions between the first and second author and may have resulted in rating a gamification component as “partially applied.”

In addition to listing individual gamification components, the applied taxonomy groups individual gamification components into five categories: system design (seven components), challenges (three components), rewards (six components), social influences (eleven components), and user specifics (four components). The original publication provides comprehensive definitions of each category [22].

#### R4: App Quality

Four of the authors (PT, RJ, JK, and FB) participated in rating app quality by means of the MARS framework [24]. However, the ratings of the first and second author were averaged due to their close cooperation in the rating process, thereby preserving independency of ratings. The MARS framework contains 19 items on a 5-point Likert scale (1=inadequate, 2=poor, 3=acceptable, 4=good, and 5=excellent) to assess app quality. Items are grouped into four subscales: engagement (5 items), functionality (4 items), aesthetics (3 items), and information quality (7 items). The average of the four scales determines the app quality score.

One item from the information quality scale requires a literature search regarding clinical efficacy. PubMed and Google Scholar were searched using the following term: [%app name%] AND [“randomized controlled trial”/”RCT”] OR [“study”]. Literature inclusion criteria were published in a peer-reviewed journal, clearly defined outcome construct that is a valid measure for asthma (eg, peak flow values) and description of the applied study design.

Additionally, MARS contains a subjective quality scale that measures the subjectively perceived app quality. This scale consists of four 5-point Likert scale questions regarding personal recommendation, potential future usage, willingness to pay for an app, and an overall star rating.

MARS is an objective (intraclass correlation coefficient [ICC] between raters=.79) and highly reliable scale (alpha=.90). As recommended by the authors of MARS [24], all raters participated in a workshop regarding the application of the framework. This workshop was based on the MARS training video [31].

### Statistical Methods

To assess the status quo of app potential in asthma apps, ratings were analyzed descriptively. ICC between raters were computed as a measure for interrater reliability of the MARS scales. Finally, correlations between the aggregated framework scores were calculated.

## Results

### General App Characteristics

Out of the 38 reviewed apps, 13 apps were available for both iOS and Android (34%). The remaining apps were exclusively developed for either iOS (16/38, 42%) or Android (9/38, 24%). The vast majority of apps was free of charge (36/38, 95%). In-app purchases were offered in seven apps (18%, 7/38). Additionally, eight apps contained some form of advertising (21%, 8/38).

Most apps were developed in the United States (14/38, 37%), followed by Switzerland (4/38, 11%), Australia, India, Spain, the United Kingdom (each 2/38, 5%), Germany, Portugal, Lithuania, Latvia, and the Czech Republic (each 1/38, 3%). Private companies accounted for the development of half of the apps (n=19), independent developers for nine apps (24%, 9/38), and university hospitals and health-related foundations for four apps each (11%, 4/38). Both nonprofit organizations and governmental nonprofit bodies were responsible for one app each (3%, 1/38). Almost half of the apps claimed that they involved health care entities in the development process (16/38, 42%).

**Table 1:** Overview general app characteristics.

App characteristics	n (%)	Mean (SD^a^)	Median (IQR^b^)	Range
Paid apps and price (USD)	2 (5)	3.49 (2.12)	3.49 (1.50)	1.99-4.99
Average user rating (iOS)	15 (39)	3.53 (0.90)	3.50 (1.00)	1.50-5.00
Average user rating (Android)	22 (58)	3.94 (0.51)	3.90 (0.50)	3.00-5.00
Number of ratings (iOS)	29 (76)	22.52 (58.26)	5 (16)	0-304
Number of ratings (Android)	23 (61)	47.35 (87.72)	17 (48.50)	0-419
Number of downloads (Android)^c^	23 (61)	5275.43 (8189.73)	3000 (4725)	30-30,000
Days since last update	38 (100)	498.63 (601.65)	177 (922)	2-2231

^a^SD: standard deviation.

^b^IQR: interquartile range.

^c^The Google Play store reports download numbers in ranges (eg, “10-50”). These values were standardized by calculating the mean of the minimum and maximum (eg, 10-50 downloads equals 30 downloads).

In general, a wide range in the distinct app characteristics became apparent (Table 1). The range was particularly noticeable in the category “days since last update”: one app was updated just two days before the review deadline (“e-symptoms”), whereas “Asthma Journal Free” was last modified over 6 years ago. On average, apps were not updated for approximately more than 1 year and 4 months.

### Evaluating App Potential

#### R1: App Functions

Tracking functions were the most common function category. Out of all 38 apps, 30 apps (79%) offered at least one of the following functions: peak flow tracking (23/38, 61%), medication tracking (22/38, 58%), symptom tracking (19/38, 50%), trigger tracking (18/38, 47%), notes (15/38, 39%), clinical asthma questionnaires (14/38, 37%), sleep tracking (11/38, 29%), health parameter tracking (9/38, 24%), medical appointment tracking (8/38, 21%), location tracking (6/38, 16%), asthma attack tracking (4/38, 11%), accomplishment tracking (3/38, 8%), snapshots (3/38, 8%), score tracking of games and quizzes (2/38, 5%), tracking through Apple or Google Health (2/38, 5%), and medication package size tracking (1/38, 3%). Except for apps using Apple or Google Health for physical activity tracking and location tracking over the global positioning system (GPS), all parameters had to be entered manually. Additionally, 26 apps (68%, 26/38) provided functions to record the tracked values over time through diaries (eg, calendars) and logs (eg, lists and charts).

A total of 26 apps (68%, 26/38) incorporated at least one of the following information functions: therapeutic information or instruction (22/38, 58%), general asthma information (20/38, 53%), medication information or instruction (16/38, 42%), first aid information or instruction (10/38, 26%), inhaler technique guidance (10/38, 26%), app tutorial (9/38, 24%), peak expiratory flow information or instruction (9/38, 24%), news (8/38, 21%), air quality (7/38, 18%), information through doctor dashboard (4/38, 11%), information through games or quizzes (3/38, 8%), and clinic or pharmacy locator (2/38, 5%).

Assessment functions can be considered an extension of tracking functions by providing an interpretation or evaluation of recorded values. Twenty apps (53%, 20/38) offered one or more of these functions: decision support (14/38, 37%), peak flow classification (9/38, 24%), adjustable asthma action plan (5/38, 13%), standardized asthma action plan (4/38, 11%), and symptom forecast (1/38, 3%).

The least common category was notification functions. A total of 18 apps (47%, 18/38) notified the user in at least one of the following contexts or events: medication intake (12/38, 32%), asthma questionnaire reminder (7/38, 18%), peak flow reminder (7/38, 18%), alert notification to health care professionals (7/38, 18%), medical appointment reminder (6/38, 16%), news notification (4/38, 11%), adjustable task reminder (3/38, 8%), weather and pollen notification (1/38, 3%), and medication package depletion (1/38, 3%).

In summary, the most widespread functions in asthma apps were tracking and information functions. More specifically, providing therapeutic information or instruction, general asthma information, as well as tracking of peak flow values, medications and symptoms were implemented by at least half of all apps.

#### R2: Potential to Change Behavior

On average, apps applied 7.12 (standard deviation [SD]=4.46) of the 26 behavior change techniques (B1-B26). The amount of applied techniques ranged from 1 (“Asthma Treatment”) to 19 (“Wizdy Pets”).

**Figure 2:**
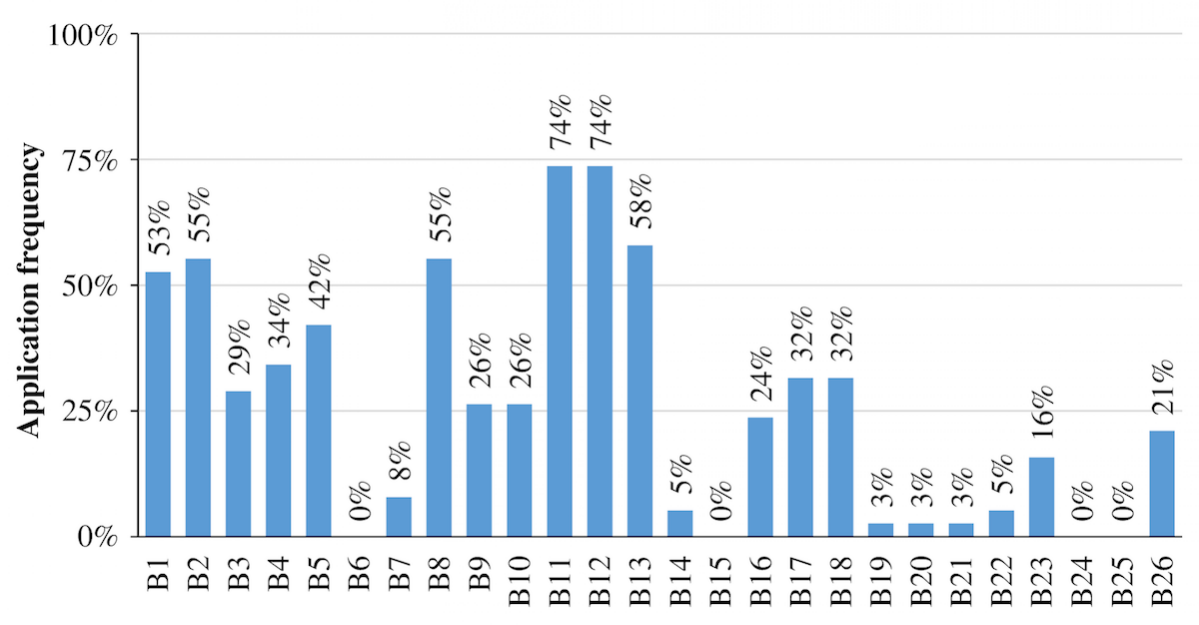
Percentage of asthma apps fully applying the corresponding behavior change technique (B1-B26; N=38).

The fraction of behavior change techniques rated as “partially applied” was negligible (27/988 ratings, 2.7%). For the sake of simplicity and interpretability, only behavior change techniques rated as “fully applied” are reported in this section and in Figure 2. The prevalence of behavior change techniques in asthma apps varied considerably between techniques. To aggregate findings, behavior change techniques were divided into four categories based on their application frequency: no application at all (ie,. technique not applied by any app resulting in an application frequency of 0%), seldom applied (ie, technique applied by one to nine apps resulting in an application frequency between 0-25%), considerable application rates (ie, technique applied by ten to 19 apps resulting in an application frequency between 25-50%), and frequent application (ie, technique applied by 20 to 28 apps resulting in an application frequency between 50-75%).

Four behavior change techniques were not applied at all: provide general encouragement (B6), teach to use prompts or cues (B15), stress management (B24), and motivational interviewing (B25).

The nine seldom applied techniques were set graded tasks (B7; 3/38, 8%), provide contingent rewards (B14; 2/38, 5%), agree on behavioral contract (B16; 9/38, 24%), provide opportunities for social comparison (B19; 1/38, 3%), plan social support or social change (B20; 1/38, 3%), prompt identification as a role model (B21; 1/38, 3%), prompt self-talk (B22; 2/38, 5%), relapse prevention (B23; 6/38, 16%), and time management (B26; 8/38, 21%). Seven techniques were applied with considerable frequency: provide information about other’s approval (B3; 11/38, 29%), prompt intention formation (B4; 13/38, 34%), prompt barrier identification (B5; 16/38, 42%), model or demonstrate the behavior (B9; 10/38, 26%), prompt specific goal setting (B10; 10/38, 26%), prompt practice (B17; 12/38, 32%), and use follow-up prompts (B18; 12/38, 32%).

The list of the most commonly applied behavior change techniques in mHealth asthma apps contained six techniques: provide information about behavior-health link (B1; 20/38, 53%), provide information on consequences (B2; 21/38, 55%), provide instructions (B8; 21/38, 55%), prompt review of behavioral goals (B11; 28/38, 74%), prompt self-monitoring of behavior (B12; 28/38, 74%), and provide feedback on performance (B13; 22/38, 58%).

#### R3: Potential to Promote App Use

**Figure 3:**
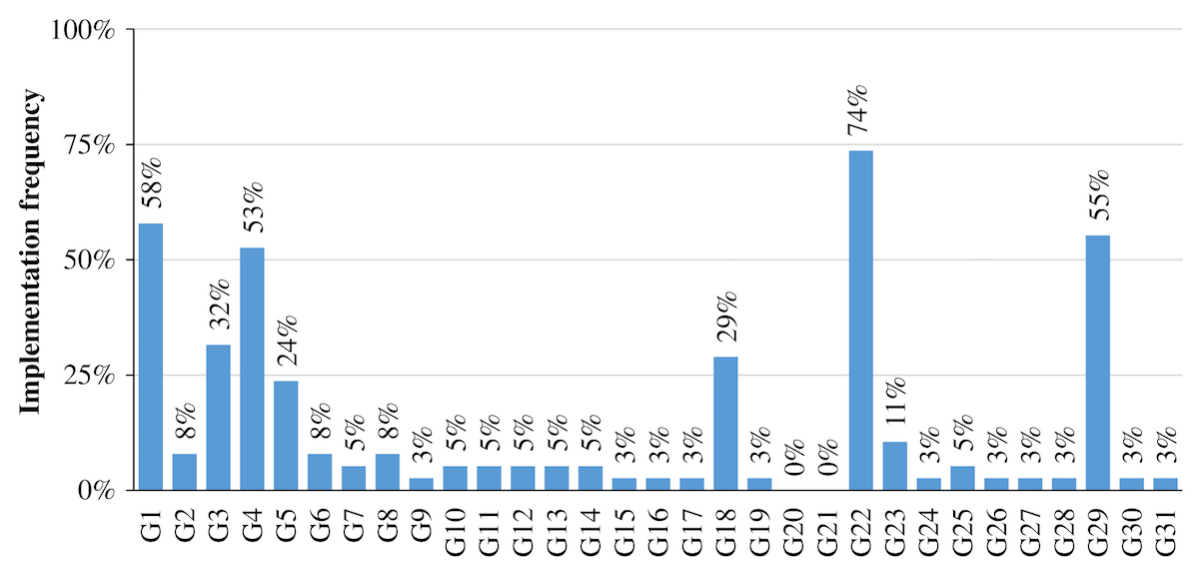
Percentage of asthma apps fully applying the corresponding gamification component (G1-G31; N=38).

Asthma apps contained on average 4.89 gamification components (SD=4.21) of a total of 31 components (G1-G31). The app with the most implemented gamification components was “WizdyPets” (n=24.50), whereas “choO” integrated the fewest components (n=0.50). Similar to the ratings of behavior change techniques, the amount of gamification components rated as “partially applied” can be considered insignificant (31/1178 ratings, 2.6%). Following the reporting logic from the previous section, only gamification components rated as “fully applied” are illustrated in Figure 3 and covered in this section.

Again, gamification components were grouped based on their prevalence into four categories: not implemented at all (ie, component was not implemented by any app resulting in an implementation frequency of 0%), seldom implemented (ie, component was implemented by one to nine apps resulting in an implementation frequency between 0-25%), considerably often implemented (ie, component was implemented by ten to 19 apps resulting in an implementation frequency between 25-50%), and frequently implemented (ie, component was implemented by 20 to 28 apps resulting in an implementation frequency between 50-75%).

In total, four gamification components were frequently implemented in the reviewed apps: feedback (G1; 22/38, 58%), meaning (G4; 20/38, 53%), shadowing (G22; 28/38, 74%), and ideological incentives (G29; 21/38, 55%).

Two gamification components were considerably often implemented: reminder (G3; 12/38, 32%) and collaboration (G18; 11/38, 29%).

However, the vast majority of gamification components was seldom implemented: audible feedback (G2; 3/38, 8%), interaction concepts (G5; 9/38, 24%), visually resembling existing games (G6; 3/38, 8%), fantasy (G7; 2/38, 5%), goals (G8; 3/38, 8%), time pressure (G9; 1/38, 3%), progressive disclosure (G10; 2/38, 5%), ownership (G11; 2/38, 5%), achievement (G12; 2/38, 5%), point system (G13; 2/38, 5%), badges (G14; 2/38, 5%), bonus (G15; 1/38, 3%), loss aversion (G16; 1/38, 3%), status (G17; 1/38, 3%), reputation (G19; 1/38, 3%), social facilitation (G23; 4/38, 11%), conforming behavior (G24; 1/38, 3%), leaderboards (G25; 2/38, 5%), altruism (G26; 1/38, 3%), virtual goods (G27; 1/38,3%), user levels (G28; 1/38, 3%), virtual characters (G30; 1/38, 3%), and self-expression (G31; 1/38, 3%). Competition (G20) and envy (G21) were not implemented in the reviewed apps.

**Figure 4:**
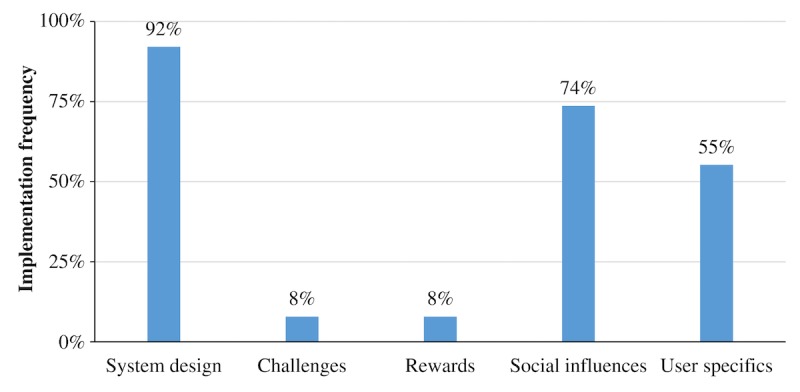
Percentage of apps which applied at least one component fully from the corresponding gamification component category.

In addition to listing individual gamification components, the taxonomy groups individual gamification components into five categories: system design, challenges, rewards, social influences, and user specifics [22]. Figure 4 illustrates how many apps implemented at least one component of the corresponding category.

**Table 2:** Descriptive results of Mobile Application Rating Scale (MARS) scores (N=38).

MARS scales	ICC^a,b,c^	Mean (SD^d^)	Range
Engagement	.92	2.77 (0.78)	1.00-4.73
Functionality	.72	3.55 (0.57)	1.75-4.42
Aesthetics	.72	3.12 (0.68)	2.00-4.78
Information quality	.88	3.24 (0.66)	1.40-4.33
Subjective quality	.85	2.65 (0.87)	1.00-4.50
App quality	.88	3.17 (0.58)	1.54-4.55

^a^ICC: intraclass correlation coefficient.

^b^Intraclass correlations were calculated as a measure for interrater reliability in a two-way random model evaluating consistency among the three sets of ratings.

^c^Interrater reliability was assessed based on the ratings of 37 apps. One app (“Asthma”) was removed from the app store before it was rated by all raters.

^d^SD: standard deviation.

In terms of gamification component category, system design components (eg, feedback, reminder, and meaning) were implemented by almost every app (35/38, 92%). The majority of apps also made use of components from the categories social influences (28/38, 74%; eg, leaderboards, competition, and reputation) and user specifics (21/38, 55%; eg, user levels, virtual character, and self-expression). In contrast, challenges (ie, goals, time pressure, and progressive disclosure) and rewards (eg, achievements, point system, and badges) were applied very infrequently (each 3/38, 8%).

#### R4: App Quality

The average quality of asthma apps can be considered marginally above acceptable (mean=3.17), whereas average subjective app quality laid between poor and acceptable (mean=2.65). In terms of MARS subscales, asthma apps performed worst in user engagement (mean=2.77) and best in app functionality (mean=3.55). Ratings for aesthetics (mean=3.12) and information quality (mean=3.24) were rather mediocre (Table 2).

In the literature review, no study was identified that evaluated the efficacy of any of the included asthma apps. The only peer-reviewed papers to mention apps were app reviews or clinical communications (eg, [32]). Hence, the information quality mean was calculated from a maximum of 6 items. Additionally, eleven out of the 38 apps (29%) could not be assessed in some of the information quality items due to a lack of corresponding functions (eg, no visualized information was provided that made it impossible to rate the visual information quality).

Considerable quality differences between apps emerged in the analysis. The range of app quality ratings and subjective app quality ratings reached from inadequate or poor (eg, “Asthma” scored a 1.54 and a 1.00 in terms of app quality and subjective app quality) to almost excellent (eg, “Wizdy Pets” scored a 4.55 and 4.50 in terms of app quality and subjective app quality).

#### Summary of Requirements (R1-R4)

Overall, the results demonstrate vast differences between apps in all of the investigated requirements of app potential (Table 3; see Multimedia Appendix 3 for the complete aggregated ranking table).

To synthesize the findings, associations between the different review frameworks were considered by calculating the corresponding correlation coefficients between the aggregated scores (Table 4). All correlations between review frameworks were significant. This pattern of correlations implies consistency of ratings between different review frameworks. In other words, apps that scored well in one review framework tended to also score well in all the other frameworks.

**Table 3:** Summary of all rated app potential requirements. Behavior change techniques or gamification components rated as “fully applied” and “partially applied” were included in the analysis.

App potential requirements		Mean (SD^a^)	Range	Scale
**R1: functions**				
	Tracking functions	4.21 (3.39)	0-11.00	0-16.00
	Information functions	3.21 (2.63)	0-8.00	0-12.00
	Assessment functions	0.87 (0.98)	0-3.00	0-5.00
	Notification functions	1.26 (1.65)	0-5.00	0-5.00
	Functions (overall)	9.55 (5.73)	1.00-24.00	0-42.00
R2: behavior change techniques		7.12 (4.46)	1.00-19.00	0-26.00
**R3: gamification components**				
	System design	2.20 (1.35)	0.50-4.00	0-7.00
	Challenges	0.22 (0.60)	0-3.00	0-3.00
	Rewards	0.26 (1.07)	0-6.00	0-6.00
	Social influences	1.37 (1.24)	0-5.00	0-11.00
	User specifics	0.84 (0.74)	0-4.00	0-4.00
	Gamification components (overall)	4.89 (4.21)	0.50-24.50	0-31.00
**R4: MARS^b^ scales**				
	Engagement	2.77 (0.78)	1.00-4.73	1.00-5.00
	Functionality	3.55 (0.57)	1.75-4.42	1.00-5.00
	Aesthetics	3.12 (0.68)	2.00-4.78	1.00-5.00
	Information quality	3.24 (0.66)	1.40-4.33	1.00-5.00
	Subjective quality	2.65 (0.87)	1.00-4.50	1.00-5.00
	App quality	3.17 (0.58)	1.54-4.55	1.00-5.00

^a^SD: standard deviation.

^b^MARS: Mobile Application Rating Scale.

**Table 4:** Pearson correlations between aggregated review frameworks (N=38). All ratings of behavior change techniques and gamification components were included in the analysis (including techniques and components rated as “partially applied or implemented”).

Aggregated review frameworks	Number of functions (R1)	Number of behavior change techniques (R2)	Number of gamification components (R3)
Number of behavior change techniques (R2)	*r*=.73 (*P*<.001)		
Number of gamification components (R3)	*r*=.33 (*P*=.04)	*r*=.80 (*P*<.001)	
MARS^a^ app quality (R4)	*r*=.38 (*P*=.02)	*r*=.56 (*P*<.001)	*r*=.62 (*P*<.001)

^a^MARS: Mobile Application Rating Scale.

## Discussion

### Principal Findings

This review analyzed whether asthma apps have the potential to improve a patient’s asthma self-management. For this purpose, different requirements of app potential were considered, namely available functions (R1), applied behavior change techniques (R2), implemented gamification components (R3), and general app quality (R4).

In terms of available functions (R1), asthma apps offered functions associated with active ingredients of effective self-management to a considerable extent (ie, asthma education, self-monitoring of symptoms or peak flow values, regular review of treatment, and an action plan [8]). However, with regard to self-monitoring of symptoms and peak flow values, not a single app offered options to track asthma-related parameters through mobile phone sensors. Without exploiting the full potential of today’s technology (eg, through automated tracking functions [33]), therapeutic advantages of mobile phones cannot be fully realized (eg, delivery of just in time adaptive interventions [34]).

The investigated apps applied a number of behavior change techniques (R2). This finding implies that the apps could in principle enable behavior change relevant for asthma self-management. However, techniques related to stress management and motivational interviewing were not implemented at all. This is particularly striking because stress management is recommended by asthma guidelines [23], and asthmatics tend to trivialize their disease symptoms [35], which might result in a lack of long-term motivation for disease management.

In general, asthma apps also seemed to be able to motivate users through gamification components (R3) but with considerable differences between apps: the relatively high standard deviation of gamification components relative to its mean and its wide range imply that only a few apps have implemented gamification features to a significant extent. For example, the category “rewards” was rarely implemented: only three out of all 38 apps (8%) implemented at least one gamification component of this category. This is somewhat surprising as rewards usually belong to the most commonly implemented gamification components [22]. Point systems, badges, and achievements can be implemented comparatively easy, and their effectiveness is backed by operant conditioning theory, one of psychology’s most recognized theories [36].

Furthermore, app quality varied widely (R4). In terms of MARS subscales, engagement scored worst. This may call into question the long-term engagement with asthma apps, a crucial factor for determining their value [7]. In other domains like diabetes self-management, researchers have observed that long-term engagement of app users is generally limited [37]. However, chronic diseases like asthma require long-term self-management [23]. One potential way to improve long-term engagement, which has been successfully applied to physical activity, are interactions with virtual coaches [38]. Thus, developers of upcoming asthma apps might consider the implementation of virtual coaches (eg, chat bots) to enhance long-term engagement.

In summary, great variation in all of the investigated requirements of app potential was found. Therefore, an unequivocal conclusion whether currently available asthma apps have the potential to improve asthma self-management is neither reasonable nor possible. However, due to the high correlations between review frameworks, this review has shown that ratings are consistent across the four requirements of app potential. This implies that high quality apps tend to score well in all requirements for app potential. For example, “Asthma Health by Mount Sinai,” which was ranked second best out of all 38 apps in terms of MARS app quality, performed also very well in terms of behavior change techniques (rank 2/38), gamification components (rank 4/38), and available functions (rank 3/38). On the other hand, one of the apps with the lowest MARS app quality ratings (“Asthma Treatment”; rank 36/38) also scored extremely poorly in terms of behavior change techniques (rank 38/38), gamification components (rank 37/38), and available functions (rank 38/38). The consistency between frameworks facilitates the decision which asthma apps to recommend to patients: a promising decision strategy might be to pick one of the best rated apps (see Multimedia Appendix 3 for aggregated app ratings); no trade-off between app potential requirements is necessary. In this way, the asthma apps with the potential to improve a patient’s self-management can be selected for clinical practice.

### Limitations

In the definition of app potential used in this paper, no measure for long-term engagement was included. As mentioned before, long-term engagement is highly relevant for asthma apps in specific and mHealth apps in general. To the best of the authors’ knowledge, there is no valid proxy that predicts long-term use reliably. App reviews and developers would highly benefit from the identification of such proxies, making it a promising field for future research.

Furthermore, apps that are not publicly available were excluded. This limits the generalizability of the results. For example, two apps, which were restricted to study participants and thus were not publicly available, have indicated promising results regarding efficacy [9,12]. Excluding not publicly available apps might have led to a systematic selection bias against apps with a greater potential for improving asthma self-management.

Finally, this review did not contain a content analysis. It only assessed whether a function was implemented, but conclusions regarding its medical value are beyond the scope of this review. However, such an analysis was already conducted in prior reviews [14,15].

### Comparison With Prior Work

In general, this review extends the findings of previous reviews by focusing on the potential of apps for asthma self-management. Some of the findings are in line with prior research [13-15,39]: the biggest concern regarding currently available apps is the lack of clinical validation. Not a single study demonstrating efficacy for any of the investigated apps could be identified. However, as argued above, this constraint might be limited to apps that are publicly available. Nevertheless, this review suggests that even without scientific proof for efficacy, at least some of the investigated apps still have the potential to improve asthma self-management.

Therefore, a somewhat more positive conclusion regarding the value of asthma apps can be cautiously drawn in comparison with prior reviews (eg, [14,15]). Among other factors, this may be due to the bottom-up research perspective focusing on app potential instead of an app’s evidence base and the development that asthma apps have undergone since the last reviews have been conducted.

### Conclusions

In conclusion, this review has found that the potential of asthma apps for improving asthma self-management varies considerably between apps. Physicians and asthmatics should therefore carefully read app reviews before deciding which app to recommend or to use. Additionally, currently available asthma apps do not take full advantage of today’s technology. Developers should address the research-practice gaps outlined in the discussion.

## Appendix

Multimedia Appendix 1 Exemplary rating criteria for behavior change techniques in mHealth asthma apps.

Multimedia Appendix 2 Exemplary rating criteria for gamification components in mHealth asthma apps.

Multimedia Appendix 3 Complete rating table of all 38 asthma apps containing app name, developer, tested app version, app characteristics, app functions, sum scores of behavior change techniques, gamification components, and aggregated MARS ratings.

## Abbreviations

GPS: global positioning system
ICC: intraclass correlation coefficient
IOS: iPhone operating system
IQR: interquartile range
IT: information technology
MARS: Mobile Application Rating Scale
SD: standard deviation
