# Supplementary material for: The Potential of Mobile Apps for Improving Asthma Self-Management: A Review of Publicly Available and Well-Adopted Asthma Apps
Source: JMIR Mhealth Uhealth. 2017 Aug 2;5(8):e113. doi: 10.2196/mhealth.7177 (PMC5559650; doi:10.2196/mhealth.7177)
Supplement: Multimedia Appendix 1 [file mhealth_v5i8e113_app1.pdf]

Multimedia Appendix 1

Exemplary behavior change technique rating criteria for mHealth asthma apps

ID	Behavior change technique	Application archetypes in asthma apps
B1	Provide information about behavior health link	General information about asthma such as causes, triggers, symptoms and FAQs
B2	Provide information on consequences	Information about controlled and uncontrolled asthma particularly regarding the consequences for a patient's quality of life and potential health risks
B3	Provide information about others' approval	The app user can interact with physicians and/or other app users through messages and social network features
B4	Prompt intention formation	Encourage user to engage in health promoting behaviors for asthma
B5	Prompt barrier identification	Identify asthma triggers and provide corresponding information and coping strategies
B6	Provide general encouragement	Reward or praise the user for generally trying to improve asthma (control) and for engaging with the app
B7	Set graded tasks	Personalized task and challenges set to increase asthma control
B8	Provide instruction	Instructing the user how to engage with the app and/or asthma control behavior such as emergency plans and usage of medical devices (e.g. peak-flow meter and inhaler)
B9	Model or demonstrate the behavior	Video tutorials, animations, or visualized descriptions of the instructions described in B8
B10	Prompt specific goal setting	Setting specific behavior goals to enhance asthma control and provide an adjustable asthma action plan
B11	Prompt review of behavioral goals	Display tracked asthma-related data (e.g. peak flow, medication, symptoms) and behavior goals (e.g. set in B7)
B12	Prompt self-monitoring of behavior	Enabling user to track asthma-related data
B13	Provide feedback on performance	Compare asthma control related goals to recorded asthma data and provide feedback
B14	Provide contingent rewards	Rewarding and/or praising users for accomplishing health tasks
B15	Teach to use prompts or cues	Remind users of environmental stimuli which are related to asthma behavior (e.g. second-hand smoke)
B16	Agree on behavioral contract	Setting up an adjustable action plan agreed on with a physician
B17	Prompt practice	Notifications for peak-flow tracking, medications, asthma tests, appointments, or other tasks
B18	Use follow-up prompts	Asthma notifications, but by messages and/or mails after app is no longer used
B19	Provide opportunities for social comparison	Provide the possibility for social comparison with other users through social network features

B20	Plan social support or social change	Provide peer networks through social network features and encourage users to use them for social support
B21	Prompt identification as a role model	Empower the user to serve as an role model for asthma control behavior
B22	Prompt self-talk	Diaries and journal entries about asthma topics
B23	Relapse prevention	Help users to avoid possibly risky situations for maintaining asthma control
B24	Stress management	Ease user's stress through relaxation exercises
B25	Motivational interviewing	Encourage self-motivating statements in general or in relation to asthma
B26	Time Management	Adjustable reminders for asthma tasks
