# Supplementary material for: The Potential of Mobile Apps for Improving Asthma Self-Management: A Review of Publicly Available and Well-Adopted Asthma Apps
Source: JMIR Mhealth Uhealth. 2017 Aug 2;5(8):e113. doi: 10.2196/mhealth.7177 (PMC5559650; doi:10.2196/mhealth.7177)
Supplement: Multimedia Appendix 2 [file mhealth_v5i8e113_app2.pdf]

Multimedia Appendix 2

Exemplary gamification component rating crieria for mHealth asthma apps

ID	Game mechanics and dynamics	Application archetypes in asthma apps
G1	Feedback	Comparing asthma control related goals to recorded asthma data and provide feedback
G2	Audible feedback	Any kind of audible feedback that is related to certain results or behaviors
G3	Reminder	Reminder for appointments, peak-flow tracking, medications, asthma tests, or other tasks
G4	Meaning	Providing information on asthma such as causes, triggers, symptoms and FAQs
G5	Interaction concepts	Providing an aesthetic user interface combined with a high degree of usability
G6	Visually resembling existing games	Resembling common games in a visual way
G7	Fantasy	Using fantasy elements such as fairy tales, speaking animals etc.
G8	Goals	Personalized tasks and challenges set to increase asthma control
G9	Time pressure	Creating time pressure through timers or countdowns to incentivize certain behaviors
G10	Progressive disclosure	Increasing the difficulty of asthma-related tasks continuously
G11	Ownership	Creating a feeling of ownership through functions such as quiz scores or personalized avatars
G12	Achievement	Rewarding accomplished asthma goals, e.g. with virtual gifts, new app features, or High score Rankings
G13	Point system	Measuring and comparing the users asthma-related accomplishments with numerical values
G14	Badges	Awarding batches as a sign for asthma goal accomplishments
G15	Bonus	Granting extra rewards for accomplishing a series of asthma-related goals
G16	Loss aversion	Punishing the user if planned asthma-related behavior is not performed
G17	Status	Providing the possibility for social comparison with other users through social network features
G18	Collaboration	Providing the possibility for social interaction with physicians or other users
G19	Reputation	Providing peer networks for social interaction
G20	Competition	Enabling users to challenge each other in reaching asthma-related goals
G21	Envy	Providing mechanisms of social comparison that lead to personal envy
G22	Shadowing	Giving users the ability to compare their results with previous scores such as Peak Flows rates
G23	Social facilitation	Goal-related interactions with physicians

G24	Conforming behavior	Providing peer networks, which enable peer pressure to increase or decrease asthma-related actions
G25	Leaderboards	Tracking and ranking of asthma-related accomplishments with leaderboards
G26	Altruism	Enabling users to give away virtual gifts
G27	Virtual goods	Enabling users to buy or trade virtual goods
G28	User levels	Indicating the users proficiency through different levels
G29	Ideological incentives	Information about controlled and uncontrolled asthma especially regarding the consequences for a patient’s quality of life and potential health risks
G30	Virtual character	The user is represented by a virtual character
G31	Self-expression	The user is given the ability for self-expression through customizable functions
